# Cellulose Fibers-Based Porous Lightweight Foams for Noise Insulation

**DOI:** 10.3390/polym15183796

**Published:** 2023-09-17

**Authors:** Mihai Seciureanu, Silviu-Marian Nastac, Maria-Violeta Guiman, Petronela Nechita

**Affiliations:** 1 Engineering and Agronomy Faculty in Braila, “Dunarea de Jos” University of Galati, 810017 Braila, Romania; mihai.seciureanu@gmail.com; 2 Faculty of Mechanical Engineering, “Transilvania” University of Brasov, 500024 Brasov, Romania

**Keywords:** cellulose fiber, foam-forming, surfactant, noise insulation, sound absorption, sound transmission loss

## Abstract

This paper examines effective and environmentally friendly materials intended for noise insulation and soundproofing applications, starting with materials that have gained significant attention within last years. Foam-formed materials based on cellulose fibers have emerged as a promising solution. The aim of this study was to obtain a set of foam-formed, porous, lightweight materials based on cellulose fibers from a resinous slurry pulp source, and to investigate the impact of surfactant percentage of the foam mixtures on their noise insulation characterisitcs. The basic foam-forming technique was used for sample assembly, with three percentages of sodium dodecyl sulphate (as anionic surfactant) related to fiber weight, and a standardised sound transmission loss tube procedure was used to evaluate noise insulation performance. Results were obtained as observations of internal structural configurations and material characteristics, and as measurements of sound absorption/reflection, sound transmission loss, and surface acoustic impedance. Based on the findings within this study, the conclusions highlight the strong potential of these cellulosic foams to replace widely used synthetic materials, at least into the area of practical noise insulation applications.

## 1. Introduction

In the past 10 years, the potential of foam-formed materials based on cellulose fibers (CF) has been harnessed in the pursuit of sustainable and environmentally friendly materials and has gained significant attention [1,2,3,4,5,6]. Indeed, foam-formed materials based on CF have emerged as a promising solution. By combining the inherent properties of CF with the unique characteristics of foam-forming, these materials offer a wide range of applications and demonstrate great potential in various industries [7,8,9,10,11].

The use of CF in noise insulation and soundproofing (NISp) applications combines their natural properties, sustainability, and cost-effectiveness, providing an attractive solution for creating quieter and more comfortable environments [1,2,5,6,7,10,12,13,14,15,16,17]. Below is a brief description of the advantages of using foams, particularly those based on CF, for NISp applications. (i) Foams with excellent sound absorption, including CF-based foams, possess a porous structure that effectively absorbs sound waves, reducing noise transmission; the interconnected void spaces in foams allow for the dissipation of sound energy through friction and air resistance [15,16]. (ii) Lightweight foams, including CF-based foams, are easy to handle and install; this characteristic is particularly beneficial for NISp applications as it minimizes additional weight on structures and systems [2,3]. (iii) Sustainable and environmentally friendly, CF-based foams are derived from renewable sources such as wood, making them a sustainable choice; these foams are biodegradable and can be produced from recycled materials, reducing environmental impact [1,4]. (iv) CF-based foams are typically non-toxic and safe for human health; they do not release harmful chemicals or volatile organic compounds (VOCs) into the environment, making them suitable for use in residential and commercial settings [6]. (v) CF-based foams have versatility and adaptability; i.e., they can be easily shaped, molded, or cut to fit various spaces and applications; this versatility allows customized solutions for NISp needs [5]. (vi) In addition to sound insulation, foams have thermal insulation properties, helping to regulate temperature and improve energy efficiency by reducing heat transfer [17]. (vii) CF-based foams can offer cost advantages compared to some synthetic alternatives; their production cost is often lower, making them a cost-effective option for NISp applications [10].

Ultra-light porous materials find significant applications in sound and energy absorption, thermal insulation, radiation shielding, and filtration [18,19,20,21,22]. Another class of materials, namely cellulose-containing materials, has gained attention in foam forming technology. In recent studies [7,8], a lightweight, highly porous, and three-dimensional, shaped, cellulose-based material called foam-paper was introduced. The production process of foam-paper shares similarities with papermaking, but offers several advantages over traditional papermaking techniques: prevention of fiber flocculation, the presence of a three-dimensional porous structure, and reduced water and energy consumption during manufacturing. Foam-paper exhibits versatility and can be applied in various fields such as insulation, packaging, filtration, and acoustics [7,9].

### 1.1. State of the Art in Materials Based on Cellulose Fibers and Foam Forming

The concept of incorporating foam into the papermaking process was initially proposed by Radvan and Gatward [23] in 1972 and later by Smith and Punton [24] in 1974, with an aim to enhance paper uniformity. Radvan and Gatward [23] utilized foam to prevent fiber flocculation caused by long fibers and high pulp suspension consistency. They introduced the Radfoam process, a foam forming technique employing a discontinuous foam forming unit connected to a small paper machine. Another study on the Radfoam process [25] demonstrated that Radfoam-made sheets exhibit a 20 to 30% higher specific volume (bulk) compared to standard handsheets. In a study by Smith et al. [26] focusing on the structure and characteristics of Radfoam-made materials, it was found that surface tension and bubble spacing within the foam significantly influence the properties of the final product, while chemical effects were deemed insignificant. Tringham [25] and Smith [26] further confirmed that Radfoam-produced material possesses improved uniformity, porosity, and strength. More recently, Al-Qararah et al. [27] from the VTT Technical Research Centre of Finland investigated the impact of various parameters on bubble size distribution in foam-formed cellulose fibers. The study revealed that increasing the rotational speed of mixing led to a decrease in average bubble size and a reduction in air content, resulting in an increased average bubble radius.

In response to the growing demand for sustainable and eco-friendly products, there has been a notable rise in interest in the reintroduction of the foaming process for the development of innovative cellulose-based materials [20,28,29,30,31]. Numerous studies have explored the creation of lightweight porous materials using nanofibrillated cellulose (NFC) through various methods [28,29,30,31]. 

In latest years, some recent studies also assessed practical aspects of foam-formed composites based on CF for insulation applications. Thus, the main purpose of the study [32] was the identification of physical, chemical, and mechanical properties of soundproofing materials. The review [33] investigated the potential of CF-based polymeric composites for automotive applications, and it concluded that cellulosic (natural) fiber-based polymeric composites offer an efficient alternative to man-made synthetic fibers within the automotive sector. Within their research, Tauhiduzzaman et al. [34] focused on the production of lignocellulosic foams using wood flour or thermo-mechanical pulp (TMP) fibers bound with cellulose nanofibrils (CNFs). Multiscale modelling was proposed to predict the mechanical properties. Hasan et al. [35] have explored various chemical modifications of lignocellulosic fiber to make it more compatible for use as reinforcement in composite materials. In addition, the applications of natural fiber composite were broadly discussed. The study [36] summarized the recent progress within the fields of preparation methods and high-performance cellulose structural material properties. The concluding remarks within the work of Xu et al. [37] suggests that thin layers of sustainable natural materials such as CF can be used to significantly improve the capability of traditional porous media to absorb noise. CF-based materials suitable for filtering, insulation, protective, and hygiene applications can be formed/obtained using aqueous foam as a carrier phase [38,39,40,41,42]. The subtle fiber−bubble interaction provides a very useful tool that can be utilized to alter both structural and mechanical material properties. Indeed, the results within the study by Ketola et al. [38] revealed that there was a clear variation in structure and strength properties between the samples made using different fibers and surfactants. Following the previous idea, the work [43] presents and discusses mechanisms that underlie the formation process, and thus, influence physical properties of formed fiber networks. Another useful conclusion of this work was that the foam rheology is affected by added fibers, which is highly important to the development of foam-forming processes.

Miranda-Valdez et al. [44] concluded that foam-formed cellulose bio-composites are a promising technology for developing lightweight and sustainable packaging materials. Their results showed that organosolv lignin enhances many properties of cellulose bio-composite foams, which are mainly required in practical applications of insulation, packaging, and cushioning. More practically, the paper [45] systematically presents and discusses the technical parameters that can be controlled in practice during foam-forming processes with cellulosic materials, in order to obtain the required properties. The focus of this analysis was on the identification of feasible solutions to compensate for decreased strength, caused by the reduced density and poor water resistance of foam-formed cellulose composites. An innovative foam/natural fiber composite was successfully developed within the work [46]. The authors demonstrated an alternative way to produce composites with promising enhanced mechanical properties. Moreover, the results presented into the study [47] can serve as a basic reference for preparation of highly stable, cellulose-based solid foams, with very good adsorption properties.

Taiwo et al. [48] have proposed an evaluation of the potential of natural fibers for building acoustics absorbers. They have concluded two main ideas: many studies have shown that sound absorbing composites based on natural fibers present good acoustic properties in a high frequency range, similar to synthetic fibers, and natural fibers have been proven as a feasible alternative to synthetic fibers for building acoustic absorbers, thereby alleviating some sustainability issues associated with synthetics.

Cellulose fibers are a natural, environmentally friendly material often used in sound insulation products. However, they do have some negative aspects when compared to other commonly used polymeric materials. Here are some of the drawbacks of using cellulose fibers in NISp applications [36,48,49,50,51,52,53,54,55,56,57,58,59]. (i) Moisture sensitivity, (ii) poor fire resistance, (iii) pest attraction, (iv) settling and decomposition, (v) dust and allergen release and (vi) limited applications mean that CFs may not be ideal for situations where moisture or fire resistance is a critical concern. (vii) While CFs are a natural and renewable resource, their production process may involve the use of chemicals and energy; thus, they have a negative environmental impact. Additionally, the need for fire-retardant treatments can add to the environmental impact. It is important to note that the choice between CFs and synthetic polymeric materials for NISp depends on specific project requirements and priorities, including cost, environmental concerns, and performance criteria. 

Within the past 10 years, researchers have focused on CF-based foams, developing and analysing different types, based on various properties of CFs and additives, and optimizing the forming process [45,58,59,60,61,62,63,64,65,66,67], in order to obtain the highest performance for practical applications of noise insulation [58,59,60,64,65,66,67], shock absorption [62], cushioning [45], packaging [45,63], and thermal isolation [61]. The investigations were mainly conducted on experimental tests [45,58,60,61,62,63,64,65,66,67], but computational simulations were also considered [45,59,62,64], modeling potential insulation characteristics compared to widely-used synthetic materials.

### 1.2. Aim of This Study

The goal of this study examined surfactant charges influence the fibrous structural configuration (fibrous network) of foam-formed cellulosic materials, as they influence sound insulation performance. In this paper, the authors have presented the experimental results for a set of foam-formed materials based on virgin, long cellulosic fibers (resinous fibers), using low-level beating, variable surfactant percentages, and a basic laboratory foam-forming technique. The results were presented and discussed in terms of noise insulation characteristics, related to fibrous network structure and specific mass. The novelty of this study is justified by: (i) a comparative analysis of noise insulation ability, in relation to the percentage of surfactant, (ii) a comparative analysis of results against those of two of the most-commonly used synthetic materials, and (iii) development of a simple and environmentally friendly formation process.

## 2. Materials and Methods

### 2.1. About Materials

#### 2.1.1. Cellulose-Based Foam-Formed Materials

The authors had developed the porous lightweight materials in a foam medium, based on virgin cellulose long fibers (resinous type fibers). The bleached softwood cellulose (typically supplying 400–800 µm average fiber length, with 15–35 µm diameter) has been used as fiber source for the foam material. Cellulose fibers with 20 °SR beating degree (according to the Shopper-Riegler method) and slurry pulp with 1.98% consistency.

The anionic surfactant used for foam forming was sodium dodecyl sulphate (SDS), commonly utilized in commercial cosmetics production. With these basic materials (slurry pulp and surfactant), three types of cellulose-based lightweight foams were obtained (please see details within Section 2.2).

#### 2.1.2. Commercial Sound Absorbers

Two different commercially available petroleum-based materials, currently used in sound insulation, were also tested, in order to provide a reference basis for analysing the performance of the CF-based foams. These materials include samples of expanded polystyrene (EPS) and extruded polystyrene (XEPS) (ISOVER, Saint-Gobain Construction Products SA (Pty) Ltd.), with specific masses presented in Section 3.1.

### 2.2. Methods Used for Forming Samples

#### 2.2.1. Foam-Forming Method

**Figure 1:**
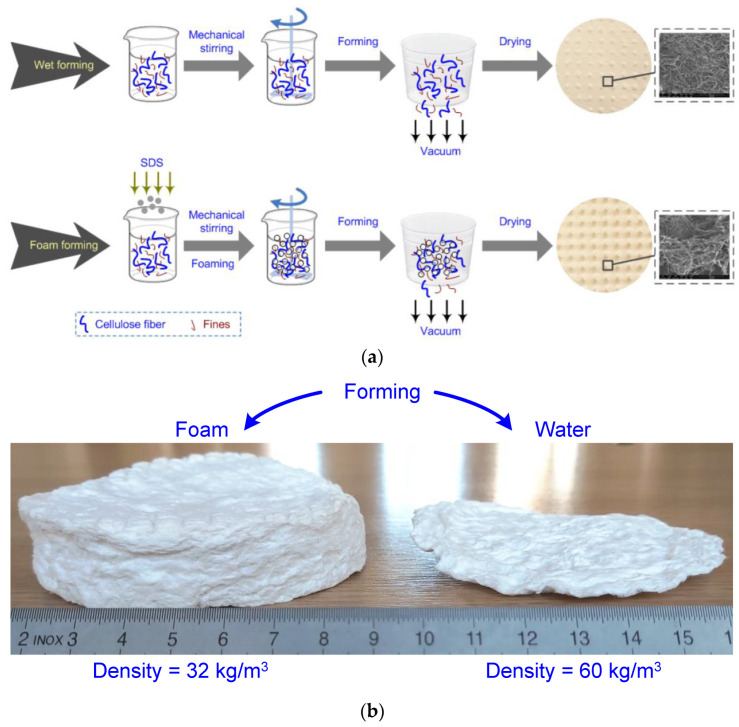
Differences between water- and foam-forming techniques, in terms of schematic diagrams [45] (**a**) and an example of final products (**b**).

Foam-formed materials based on cellulose fibers consist of a matrix of cellulose fibers embedded within a foam structure. Cellulose fibers, derived from plant sources such as wood, cotton, or hemp, serve as the reinforcing material. The manufacturing process involves several stages, starting with the disintegration of the cellulose fibers into a water suspension. This suspension is then mixed with foaming agents and additives to create a foam-like structure. Subsequently, the foam is consolidated and dried to form a solid material. Diagrams and pictures in Figure 1 present the main difference between water- and foam-forming techniques, highlighting clearly the advantages of the last procedure.

The foam-forming technique is briefly described. The cellulose fibers (resinous virgin cellulose fibers) with a pre-determined weight to obtain a slurry pulp with 1.98% consistency were soaked overnight (approx. 24 h) using distillate water with 1% sodium hydroxide (1 N concentration). 

**Figure 2:**
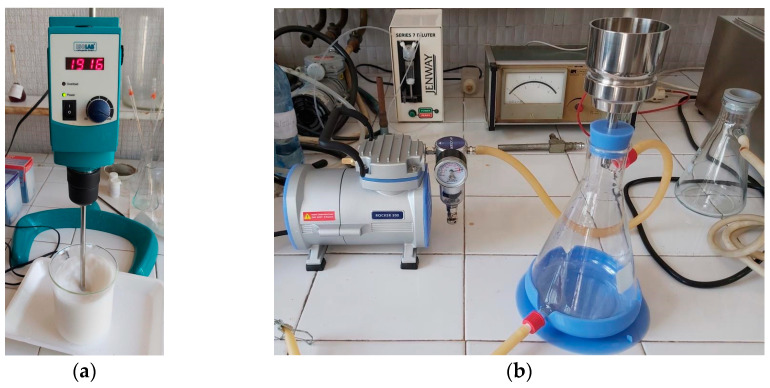
Equipment used in foam-forming procedure: (**a**) Mixer for homogenization of slurry pulp and SDS; (**b**) Buchner funnel with vacuum pump for dewatering.

Next, the slurry pulp was mixed at high shear velocity (up to 2200 rpm) for 20 min, in order to increase the porosity of final foam structure by air entraining (Figure 2a). During the agitation process, a controlled percentage of surfactant (relative to the pulp weight) was added, to produce foam. The authors proposed to analyse three types of materials with various air contents. Hence, three different quantities of surfactant were used as follows: 2%, 4%, and 6%, relative to the fiber weight.

The mixture of foam and fibers in suspension were filtered and dewatered, using a Buchner funnel (Figure 2b), with sample holder diameter correlated to the requirements of equipment used in experimental investigations (e.g., 100, 72 and 28.5 mm). A filter paper was used at a sample bottom, in addition to the filtering system of the Buchner funnel, in order to obtain a sample surface as flat as possible. The filtering was developed at a low level of vacuum, for approx. 20 min, to insure a suitable dewatering, while maintaining the sample structural integrity.

#### 2.2.2. Drying Methods

After dewatering, the samples of foam material were carefully transferred, to avoid destroying of their integrity, from the Buchner funnel to the drying table in a mould with a specific diameter appropriate for the equipment used in acoustical investigations. The samples were dried at room temperature (approximately 22 °C) and 50–60% relative humidity, for 24 to 48 h. 

### 2.3. Methods for Investigation of Noise Insulation Ability

The foam samples, obtained according to the previously described procedure, were analysed in order to estimate the acoustic insulation capabilities, in terms of absorption/reflection coefficients, sound transmission loss, and acoustic impedance. All parameters were evaluated at normal incidence. 

**Figure 3:**
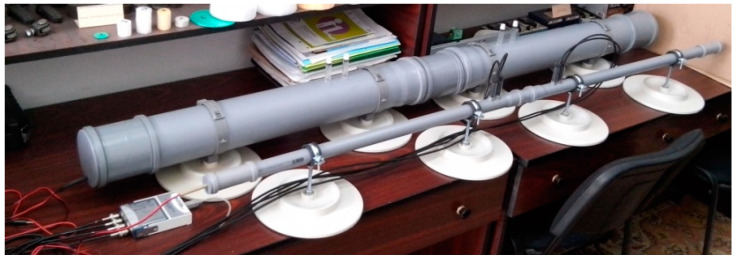
Transmission loss tubes used for evaluation of noise insulation ability of foams.

Experimental investigations were conducted using a transmission-loss tube setup, containing two types of conventional acoustic tubes (Kundt tubes), for samples with 100 and 28.5 mm diameters (Figure 3). The frequency ranges for each tube are (10–2000) Hz for the large diameter tube (denoted K1 within this paper), and (100–7000) Hz for the small diameter tube (denoted K2 within this paper). Both devices enable floating anechoic termination and four-microphone setup configuration.

Based on the transfer matrix method, the “two-load” technique was adopted for all experimental investigations, which uses two different tube loads, such as “nearly–anechoic” and “free” terminations. Both acoustic tubes enable this requirement. 

The tubes include suitable holders for the PCB–130E20 ICP^®^ Electret Array Microphone (PCB Piezotronics Inc., Depew, NY, USA) acoustic transducers. Digital acquisition of acoustic signals was practically assured with the NI-USB-9233/9162^®^ (NI, Austin, TX, USA) pair devices, and managed using a specific NI-LabVIEW^®^ (NI, USA) application. A high sampling rate (25 kHz) was used, in order to provide highest accuracy of acquired signals, and enabling a consistent/conformal computational post-processing procedure. The computational developments were performed using a set of Matlab^®^ R2014b (MathWorks, USA) applications.

The measurements were developed based on the transfer function method according to ISO 10534-2 and ASTM E1050-12 international standards for absorption coefficient and ASTM E2611-17 for transmission loss [58,64,66].

## 3. Results

### 3.1. Cellulose Fiber-Based Porous Lightweight Materials

**Figure 4:**
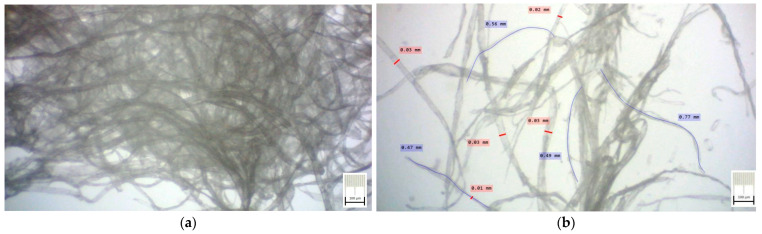
Micropgraphs of slurry pulp used for foam-forming materials: (**a**) general view; (**b**) detail view with length and diameter measurement.

The authors obtained three types of foam materials using cellulose fibers from resinous slurry pulps with 20 °SR. Optical micrographs in Figure 4 present the internal fibrous structure of slurry pulp before starting the foam-forming process, with few marked measurements related to fiber length and diameter respectively. Photos were obtained using an optical microscopy technique, with transmitted dead white light, based on a DELTA Optical Three-Ocular Microscope model SZ-450T^®^ (Delta Optical, Minsk Mazowiecki, Poland), and Bresser MikrOkular Full HD Digital Camera^®^ (Bresser GmbH, Rhede, Germany). Each photo provides a dimensional grid gained by a standard calibration grid lamella.

Foam density is strongly related to the structure and strength of fibrous network, which is quantified as the numbers of contacts between fibers. Foam bubbles limit the possible localization of fibers, thus, resulting in more open pores, and also in more fiber contacts at areas in-between the bubbles. 

**Figure 5:**
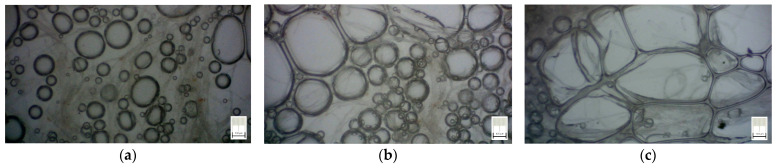
Micrographs of cellulose fibers in foam (wet samples, at the end of mixing process), for different SDS percentage: (**a**) S1 sample with 2% SDS; (**b**) S2 sample with 4% SDS; (**c**) S3 sample with 6% SDS.

Micrographs in Figure 5 reveal these facts, for the three different types of samples. It was observed that higher SDS percentage leads to larger foam-bubbles, increasing the void between fibers, and, thereby, increasing the possibility to obtain high porosity of foam. 

**Figure 6:**
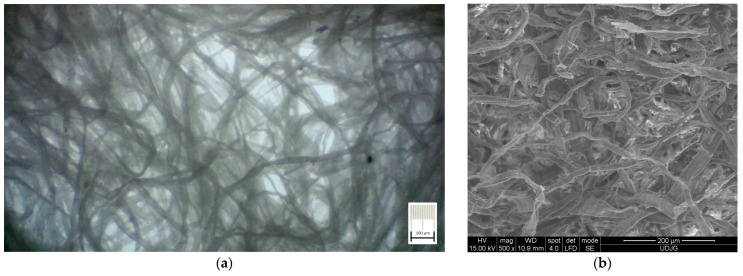
Internal fibrous structure of S1 sample at the end of drying process: (**a**) Optical micrograph; (**b**) SEM micrograph.

This latter remark will be validated through mass and density measurements. However, the micrographs presented in Figure 6, for a certain foam sample, reveal a porous, fibrous internal structure in its final material (after dewatering and drying processes) compared to the initial structure of slurry pulp (see Figure 4a). Photos in Figure 5 and Figure 6a were obtained using the same technique and equipment presented in the previous paragraph, and photo in Figure 6b was obtained through SEM technique (using available equipment at “Dunarea de Jos” University of Galati, RO).

**Figure 7:**
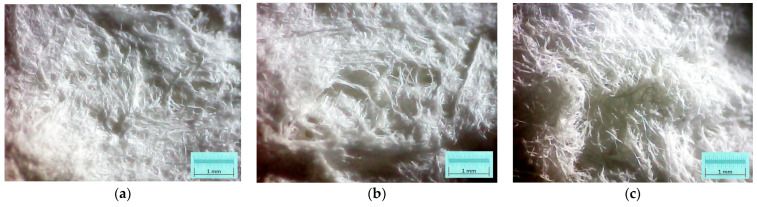
Optical micrographs of the samples external surface (dry and ready-to-use samples) for: (**a**) S1 sample; (**b**) S2 sample; (**c**) S3 sample.

For acoustical investigations using the normal incidence hypothesis, the quality of the external surface is an important aspect. Thus, in Figure 7, are depicted three micrographs related to each category of samples. It was easily observed that an improvement of surface airy porous structure is obtained with an increasing SDS percentage.

**Figure 8:**
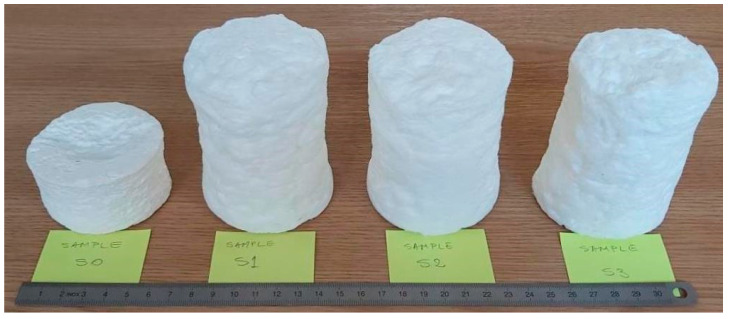
A set of samples used for noise insulation investigations.

A set of samples from each category are presented in Figure 8, where S(1,2,3) samples denote (2,4,6)% of SDS (relative to the fibres content of slurry pulp) into the initial foam-forming mixture, and S0 is the reference sample, simply obtained by water-forming procedure (without SDS addition).

The air content was computed for each sample, based on the initial volume of slurry pulp and SDS and the final volume of foamed mixture (directly evaluated after stopping of mixing process). The average values of air content, for each type of foam samples are provided in Table 1 (where STD means standard deviation).

**Figure 9:**
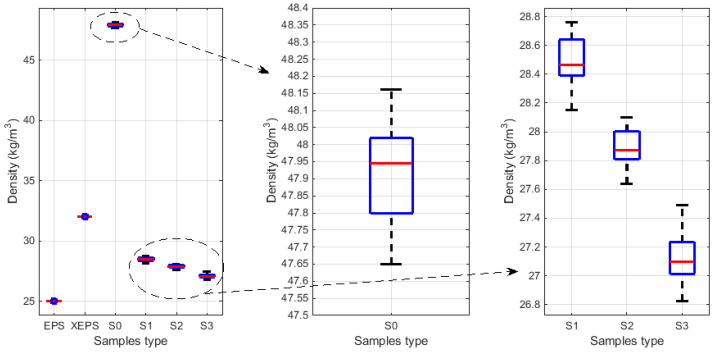
The stochastic diagram related to the samples density (general view for all samples, and details of water- and foam-formed foams respectively).

**Figure 10:**
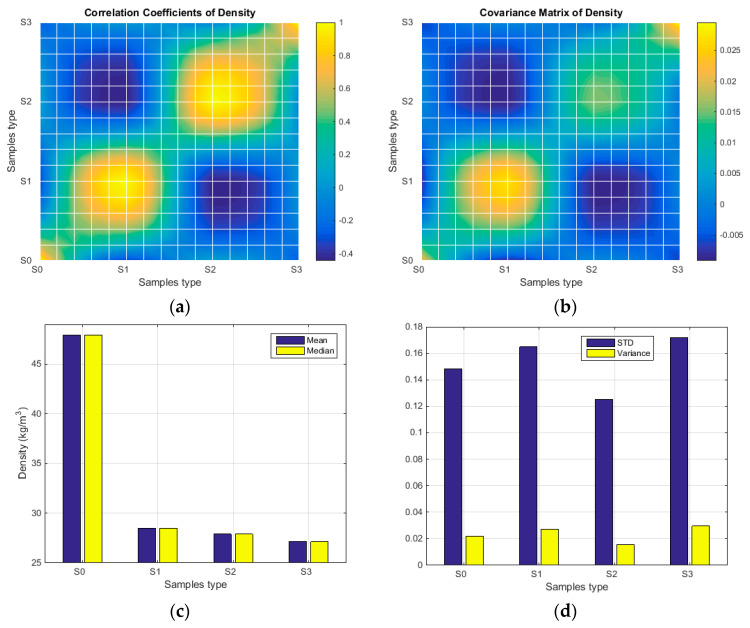
Statistical parameters of density in respect to material type: (**a**) Correlation coefficient; (**b**) Covariance matrix; (**c**) Mean and median values; (**d**) Standard deviation and variance.

**Figure 11:**
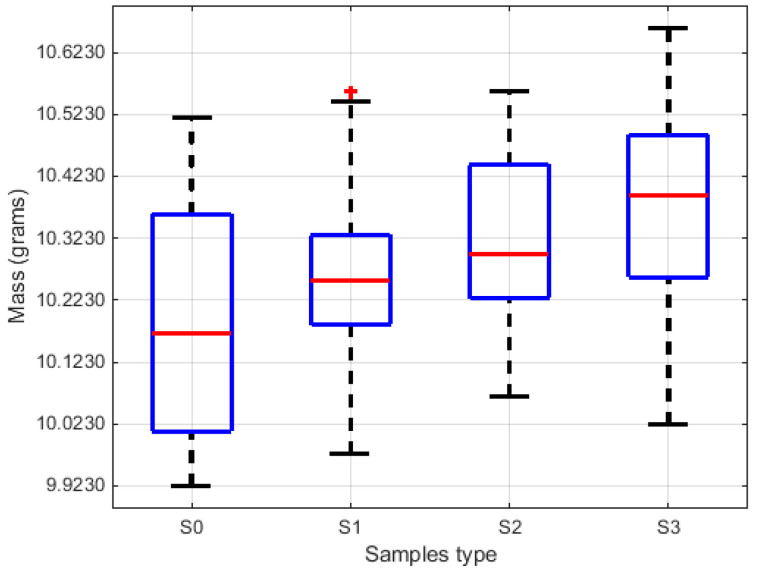
The stochastic diagram related to the samples’ mass.

**Figure 12:**
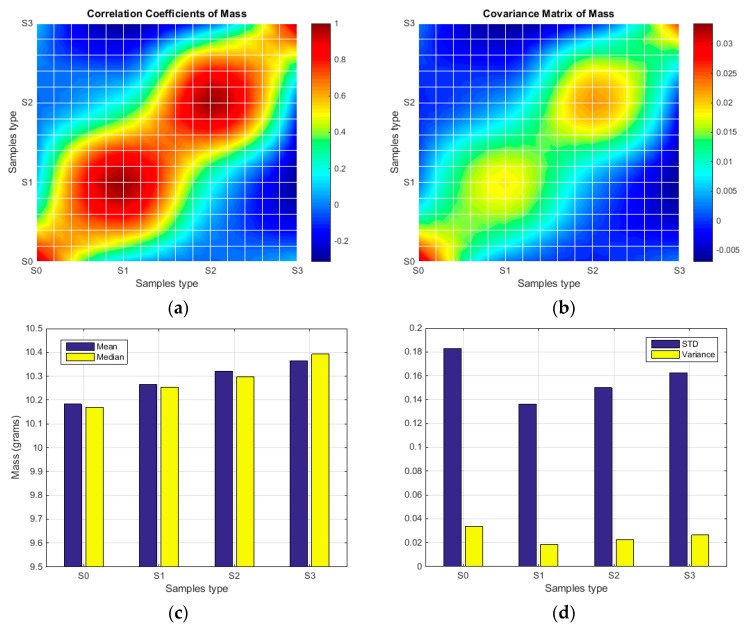
Statistical parameters of mass in respect to material type: (**a**) Correlation coefficient; (**b**) Covariance matrix; (**c**) Mean and median values; (**d**) Standard deviation and variance.

The density and mass of the foam samples were evaluated, and the values were presented in Table 2 (table contains average values for samples S0, S1, S2, and S3). A stochastical analysis was performed for each parameter, and the comparative results were provided in graphical form. The stochastic parameters for sample density are depicted in the boxplots in Figure 9, and the surface/bar plots in Figure 10 illustrate the behaviour of statistical parameters in relation to the acquired values of density. Similarly, diagrams within Figure 11 and Figure 12 show the statistical parameters in relation to the mass.

### 3.2. Noise Insulation Characteristics

**Figure 13:**
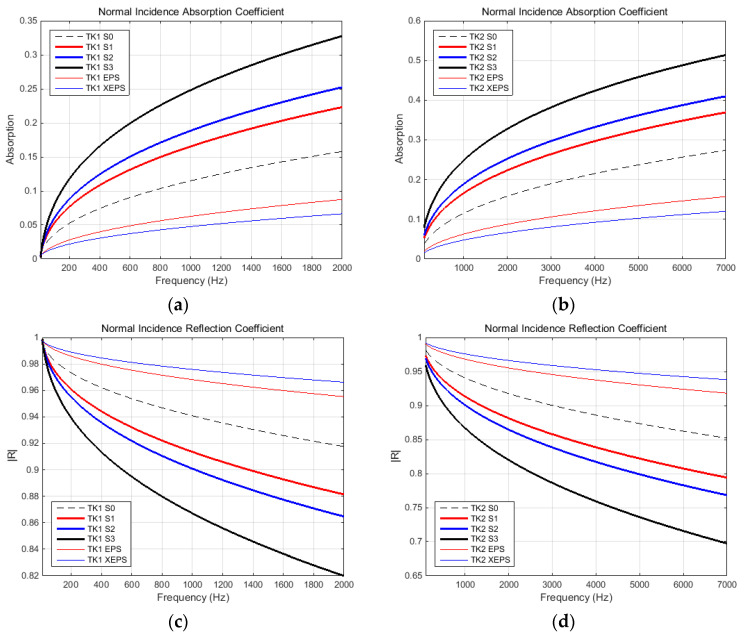
Sound absorption and reflection ability of samples at normal incidence, in terms of: Absorption coefficient recorded in tube K1 (**a**) and tube K2 (**b**) respectively; Reflection coefficient recorded in tube K1 (**c**) and tube K2 (**d**) respectively.

**Figure 14:**
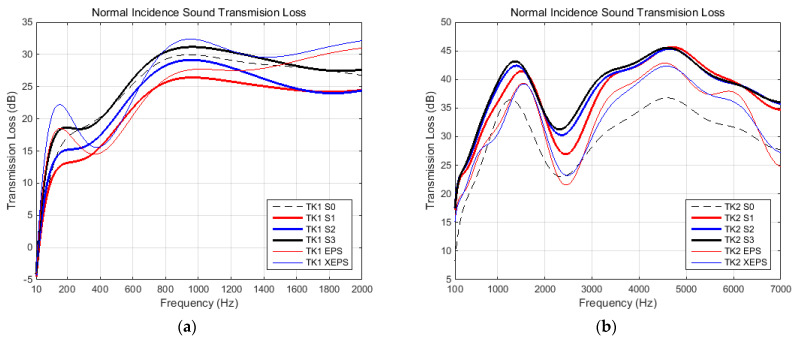
Sound transmission loss characteristic at normal incidence, recorded in tube K1 (**a**) and tube K2 (**b**) respectively.

**Figure 15:**
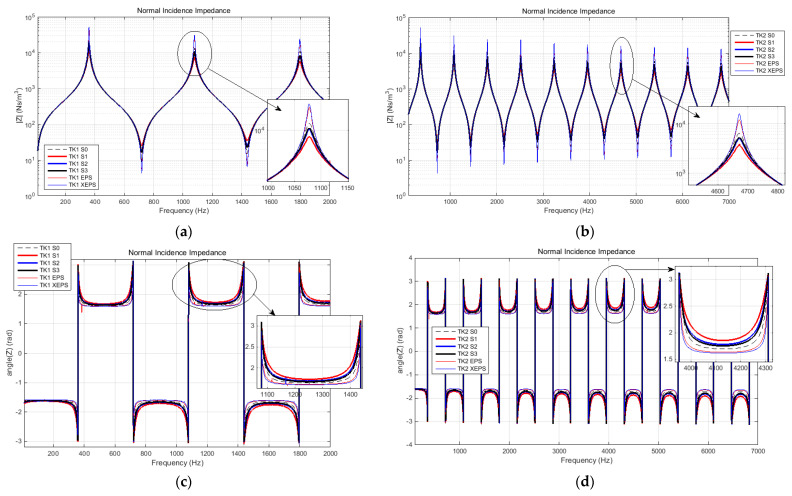
The impedance at normal incidence of sound, in terms of: impedance magnitude for signals recorded in tube K1 (**a**) and tube K2 (**b**); and impedance phase (angle) for siganls recorded in tube K1 (**c**) and tube K2 (**d**).

The noise insulation ability of proposed materials were evaluated in terms of following parameters: normal incidence absorption (α)/reflection (R) coefficients, normal incidence sound transmission loss (STL), and impedance at normal incidence (Z). Whole parameters were evaluated based on both transmission loss tubes (K1 and K2). Complex domain parameter R has been adopted only the magnitude for this study, because this is commonly presented in the technical literature related to comparative analyses regarding noise insulation capabilities of different materials. However, the Z parameter, also complex, was presented in terms of both magnitude and phase, in order to reveal the conformity of sound spectrum evaluations. All parameters were presented with respect to frequency (suitable for each tube’s capability). Thus, the diagrams were grouped as follows: absorption and reflection coefficients in Figure 13, STL in Figure 14, and impedance components in Figure 15.

**Figure 16:**
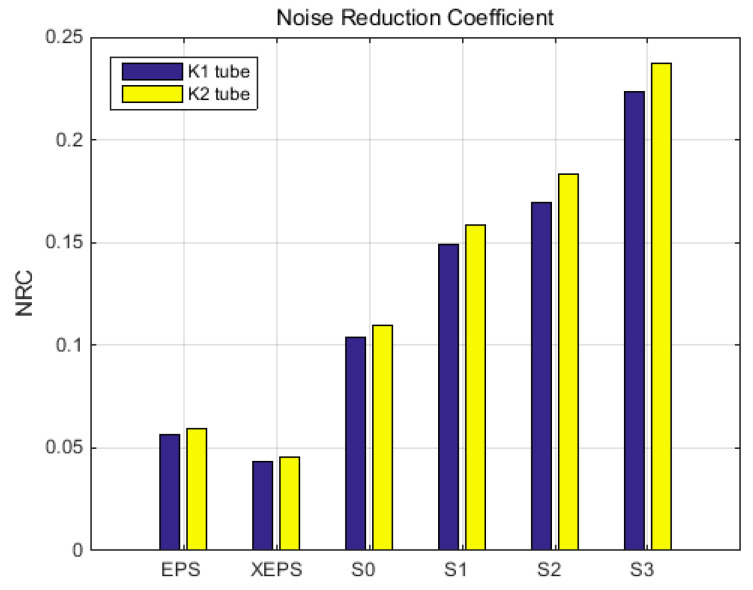
Noise reduction coefficient, average, for both transmission loss tubes (K1 and K2).

In addition to previously presented parameters, in the acoustics technical literature another indicator is typically used, which is related to noise insulation capability, particularly for middle-range frequencies. This is known as the Noise reduction coefficient (abbreviated NRC; introduced by W. R. Farrell in the early 1950s [68]), and this is represented by a single number, ranging from 0.0 to 1.0, which describes the average sound absorption performance of a material. NRC is the arithmetic average, rounded to the nearest multiple of 0.05, of the sound absorption coefficients for a specific material and mounting condition, evaluated at the octave band centre frequencies of 250, 500, 1000, and 2000 Hz. Within this study, NRC is given as raw values (unrounded) because this research was based on impedance tube for the noise absorption investigation, and authors have proposed to comparatively characterize the raw materials, not within practical dimensional and mounting conditions. The raw values of NRC, for each tube and sample type, were presented in Table 3. A graphical representation was presented in Figure 16 in order to facilitate comparative analysis. 

## 4. Discussion

The results have clearly shown a suitable foam structure for noise insulation applications; the microphotographs in Figure 5 present the initial bubble structure (wet state during the mixing process of foam-forming process) of the proposed material, with increasing percentage of SDS, inducing larger bubbles in fiber-based foam. The positive effect of the airy wet structure can be observed in the final state of samples (see optical and SEM micrographs in Figure 6) as they compare to the initial state of the slurry pulp (see Figure 4a). Moreover, the surface microphotographs presented in Figure 7 clearly reveal a porous structure for each type of material, with air content improving with increasing SDS. In addition to the wet state air content (see values in Table 1), the density values (see Table 2) support this concluding remark, with final air content percentages of 70, 74, and 80 for S1, S2, and S3, respectively. Statistical analyses (see Figure 9, Figure 10, Figure 11 and Figure 12) highlight good correlation coefficient matrices for the results, with very close mean and median values, small variances and standard deviations, and a very small covariance matrix.

Based on the results within Section 3.1, it can be stated that the initial aim of obtaining a highly porous internal structure was reached. The maximum SDS percentage was limited by sample structural stability (based on visual investigations). Systematically, through repeated tests with various SDS percentages, we have shown that the high level of air-bubbles within the pulp structure had a role in the structural stability loss of the out-of-mould sample (final stage sample, ready-to-use). The minimum SDS percentage was based on relevant air-bubbles volumes in foam mixtures (microscope-based investigations).

Previously mentioned findings were validated through noise insulation characteristics. The results within Section 3.2 validate the structural morphology-based remarks. In terms of acoustical parameters, we found that the normal incidence absorption/reflection coefficients (see diagrams in Figure 13) indicated improved absorption, but poorer reflection, with increasing SDS percentage. Even the reference sample (water-formed sample, S0) had better absorption/reflection properties than synthetic materials (EPS, XEPS). This aspect has been supported by the very open pore structures of the fiber-based materials, compared to the closed structures of EPS/XEPS. A distinct increase in absorption capacity was observed in the S3 sample (with a higher percentage of air-content), as evidenced by its very high porosity (and tortuosity) parameter. The poor reflection coefficient of the highly porous S3 sample (also the others fiber-based samples) can be explained by the surface’s rugged profile (please see micrographs in Figure 7), which induces sparse reflections, compared to the smooth surface of reference synthetic materials which enable directional reflection. It should be noted there was a good correlation between the results obtained from the two acoustic tubes (K1 and K2).

Sound transmission loss (STL) at normal incidence represents another important parameter used in sound insulation characterization. Diagrams within Figure 14 show the evolution at this parameter in respect to frequency (according to each tube’s capability). The conclusions from these results are listed here. (i) The large tube (K1) provides relevant information for low- and mid-range frequencies, while the small tube (K2) provides information for a whole significant range in noise insulation practice. (ii) Overall, the S3 sample performed better than the other foam-formed materials (S2, S1; performance level decreasing in this order). (iii) S1–S3 foam-formed materials, in comparison to synthetic references (EPS, XEPS), for low-middle range frequencies, have clearly better STL performance in the whole range. (iv) The reference, water-formed fiber-based material (S0) provided better STL performance compared to the S3 sample, but only for low-middle range frequencies; its other characteristics were poorer than those of synthetic reference materials (EPS, XEPS).

The proposed foam-formed cellulose fiber-based materials are able to provide comparable STL performances to those of widely used synthetics.

Diagrams within Figure 15 depict the impedance characteristic of sample’s surfaces, at normal incidence, in terms of magnitude and phase. The graphs were relatively identical in general shape; however, detailed views of the diagrams, particularly those of the resonance domain (randomly adopted for each graph) reveal some observations. (i) The similitude of shape for both magnitude and phase reveals a correlation of the sample geometry according to the acoustic transmission loss tube used for investigation. (ii) Detailed views provide very useful information regarding damping capability of each sample, which has a direct implication on its capacity for noise insulation. For example, it can be easily observed that the group of foam-formed samples (S1–S3) enable higher damping ratios than did the other reference samples. Within this group of samples, S1 provides better damping and S3 has the poorest dampling. This aspect can be explained by the fiber density, which is greater for S1 than S3, as the damping is supplied by fiber deformations. In contrast, the S0 sample enabled a high damping ratio compared to synthetic materials, because of its open-pore structure, which had internal mechanisms of wave propagation and pore wall (fiber-based) deformations.

Finally, the values used for NRC computing were acquired from sound absorption coefficient diagrams (see Figure 13a,b). Analyses of the numerical results in Table 3 and the graphical representation in Figure 16 show a good correlation between the two acoustic tube investigations. NRC increases with sample porosity; thus, a better NRC was provided by the foam-formed materials compared to the reference samples, especially the synthetics. The small differences between values computed for the two tubes are due to the different functional characteristics of each tube, and by the homogeneity of samples. The latter also impacts the absorption/reflection coefficients, and acoustic impedance diagrams.

## 5. Conclusions

The objectives of this research were met: Cellulose fiber-based, porous, lightweight materials were obtained using the basic foam-forming technique, and preliminary results (through material and structural characterization) have shown a suitable ability for noise insulation applications, as verified in specific acoustic investigations. The authors’ assumption that the SDS percentage influences noise insulation performances was also experimentally tested and validated. Indeed, increasing SDS charge results in an improved noise insulation capability thorough its porous internal configuration (with greater voids structure and spatial distribution). Additional results obtained during the experimental tests (e.g., dimensional and structural stability) were not directly presented into this paper (such as discussed diagrams or tables), but were obviously included within the adopted hypotheses for this study.

Foam-formed materials based on cellulose fibers present a sustainable and promising alternative to conventional materials. Leveraging the mechanical properties of cellulose fibers and the advantages of foam forming, these materials offer enhanced strength, low density, and environmental benefits. In the future, advancements in processing techniques and the development of hybrid materials using cellulose fibers with other reinforcing materials, such as natural or synthetic polymers, could further expand the range of applications and enhance the overall performance of foam-formed materials.

## Figures and Tables

**Table 1:** Average values of air content.

Sample	Inital Volume (mL)	Final Volume ^1^ (mL)	Air Content (%)	STD
S1	500	710	42	0.122
S2	500	760	52	0.141
S3	500	800	60	0.135

^1^ Average values (20 items for each sample).

**Table 2:** Average values of samples density and mass.

Sample	EPS	XEPS	S0 ^1^	S1 ^1^	S2 ^1^	S3 ^1^
Density (kg/m^3^)	25.000	32.000	47.9095	28.4846	27.8818	27.1249
Mass (×10^−3^ kg)	9.969	12.760	10.183	10.265	10.322	10.366

^1^ Average values (20 items for each sample).

**Table 3:** Noise reduction coefficients (NRCs) as raw, unrounded values.

Sample	EPS	XEPS	S0 ^1^	S1 ^1^	S2 ^1^	S3 ^1^
NRC—tube K1	0.0566	0.0432	0.1037	0.1489	0.1696	0.2231
NRC—tube K2	0.0594	0.0454	0.1099	0.1586	0.1832	0.2376

^1^ Average values (20 items for each sample).

## Data Availability

Not applicable.
